# Characterization of the Prophage Repertoire of African *Salmonella* Typhimurium ST313 Reveals High Levels of Spontaneous Induction of Novel Phage BTP1

**DOI:** 10.3389/fmicb.2017.00235

**Published:** 2017-02-23

**Authors:** Siân V. Owen, Nicolas Wenner, Rocío Canals, Angela Makumi, Disa L. Hammarlöf, Melita A. Gordon, Abram Aertsen, Nicholas A. Feasey, Jay C. D. Hinton

**Affiliations:** ^1^Institute of Integrative Biology, University of LiverpoolLiverpool, UK; ^2^Laboratory of Food Microbiology, Department of Microbial and Molecular Systems, Faculty of Bioscience Engineering, KU LeuvenLeuven, Belgium; ^3^Department of Cell and Molecular Biology, Uppsala UniversityUppsala, Sweden; ^4^Institute of Infection and Global Health, University of LiverpoolLiverpool, UK; ^5^Malawi-Liverpool-Wellcome Trust Clinical Research ProgrammeBlantyre, Malawi; ^6^Liverpool School of Tropical MedicineLiverpool, UK

**Keywords:** Gifsy, ST64B, BTP5, P22, polylysogeny, D23580

## Abstract

In the past 30 years, *Salmonella* bloodstream infections have become a significant health problem in sub-Saharan Africa and are responsible for the deaths of an estimated 390,000 people each year. The disease is predominantly caused by a recently described sequence type of *Salmonella* Typhimurium: ST313, which has a distinctive set of prophage sequences. We have thoroughly characterized the ST313-associated prophages both genetically and experimentally. ST313 representative strain D23580 contains five full-length prophages: BTP1, Gifsy-2^D23580^, ST64B^D23580^, Gifsy-1^D23580^, and BTP5. We show that common *S.* Typhimurium prophages Gifsy-2, Gifsy-1, and ST64B are inactivated in ST313 by mutations. Prophage BTP1 was found to be a functional novel phage, and the first isolate of the proposed new species “Salmonella virus BTP1”, belonging to the *P22virus* genus. Surprisingly, ∼10^9^ BTP1 virus particles *per* ml were detected in the supernatant of non-induced, stationary-phase cultures of strain D23580, representing the highest spontaneously induced phage titer so far reported for a bacterial prophage. High spontaneous induction is shown to be an intrinsic property of prophage BTP1, and indicates the phage-mediated lysis of around 0.2% of the lysogenic population. The fact that BTP1 is highly conserved in ST313 poses interesting questions about the potential fitness costs and benefits of novel prophages in epidemic *S.* Typhimurium ST313.

## Introduction

Non-typhoidal serovars of *Salmonella enterica* have typically been associated with self-limiting enterocolitis, and bloodstream infection has been an uncommon complication. However, over the past decade, it has become apparent that non-typhoidal serovars are a frequent cause of life-threatening blood stream infection in sub-Saharan Africa (SSA), a clinical syndrome known as invasive non-typhoidal *Salmonella* (iNTS) disease (Feasey et al., 2012). Malaria, HIV and malnutrition have all been strongly associated with iNTS disease which was, in fact, one of the first AIDS defining illnesses (Bottone et al., 1984; Duggan and Beyer, 1975; Feasey et al., 2012, 2015). It has recently been estimated that iNTS is responsible for ∼390,000 deaths/year in SSA (Ao et al., 2015). As a consequence of the association with immunosuppressive illnesses, it had been assumed that iNTS disease is a natural consequence of host factors and not due to intrinsically different properties of the pathogen.

Whilst a number of non-typhoidal *Salmonella* serovars have been isolated from patients with iNTS disease, *S. enterica* serovar Typhimurium (*S*. Typhimurium) is known to be the serovar most frequently associated with iNTS disease in Africa (Reddy et al., 2010). This prompted the whole genome sequencing of hundreds of *S.* Typhimurium isolates from cases of iNTS disease in SSA, and the discovery of a novel multi-locus sequence type (ST): ST313 (Kingsley et al., 2009; Okoro et al., 2012). Genomic analysis has largely focused on the representative strain D23580, suggesting that ST313 is characterized by a distinct accessory genome, a pattern of genome degradation that shared some similarities with host-restricted *Salmonella* serovars and a virulence plasmid that encodes multidrug resistance (MDR; Kingsley et al., 2009; Okoro et al., 2015).

The population structure of ST313 consists of two discrete phylogenetic groups, denoted lineage I and II. Lineage II was reported to have clonally replaced lineage I in the mid-2000s, and its subsequent dominance has been linked to acquisition of chloramphenicol resistance (Okoro et al., 2012). The strong association between ST313 and iNTS disease compared to other STs of Typhimurium, and other serovars of *Salmonella*, has led to the hypothesis that ST313 has adapted to cause systemic disease in the SSA human niche. However, despite considerable research into the effects of core genome degradation and *in vitro* phenotypes that differentiate ST313 from other clades of *S*. Typhimurium (Carden et al., 2015; Okoro et al., 2015; Ramachandran et al., 2015; Yang et al., 2015; Singletary et al., 2016), a mechanism that would confer an enhanced ability of *S*. Typhimurium ST313 to invade the human bloodstream has yet to be described (Lokken et al., 2016).

When the representative ST313 strain D23580 was initially sequenced, six prophages were identified and arbitrarily named Blantyre Prophage “BTP”1-6 (Kingsley et al., 2009). A follow-up study confirmed that this prophage repertoire is characteristic of all ST313 isolates belonging to lineage I and II (Okoro et al., 2015). Despite the striking conservation of these prophages across isolates from all over the African continent, the ST313 prophages have not been characterized experimentally. Prophages are virus genomes inserted into the bacterial chromosome. Prophages replicate concomitantly with the host genome, unless an induction signal is detected (such as host cell damage) when the prophage is able to exit the chromosome and replicate lytically as a virus. To promote host survival, and therefore the evolutionary success of the phage, prophages can carry accessory genes that contribute to host fitness. The established association between *Salmonella* virulence factors and prophages (Figueroa-Bossi et al., 2001; Ho et al., 2002; Wagner and Waldor, 2002; Brüssow et al., 2004) prompted us to explore the biology of the ST313 prophage regions.

## Materials and Methods

### Bacterial Strains and Culture Conditions

A complete list of strains and plasmids used in this study and construction details can be found in **Table 1**. Lysogeny broth (Lennox formulation; LB) contained 10 g tryptone, 5 g yeast extract and 5 g NaCl *per* liter (pH 7.0). All strains were cultured in LB broth for 16 h at 37°C with shaking at 220 rpm, unless otherwise stated. For chemical phage induction, mitomycin C (Sigma-Aldrich M4287) suspended in water was sterilized using a 0.22 μm filter and added to exponentially growing cultures (OD_600_ = 0.3) at a final concentration of 2 μg/ml. When required, LB was supplemented with the following antibiotics: ampicillin (Ap), 100 μg/ml; chloramphenicol (Cm), 25 μg/ml; kanamycin (Km), 50 μg/ml; gentamicin (Gm), 20 μg/ml; oxytetracycline (OxyTc), 10 μg/ml; tetracycline (Tc), 20 μg/ml.

**Table 1:** Bacterial strains and plasmids.

Bacterial strains or plasmids	Description^a^	Reference/origin
**Bacteria: *S.* Typhimurium**		
**D23580 derivatives**		
JH3621	D23580 WT	Kingsley et al., 2009
JH3874	ΔGifsy-1	This study
JH3875	ΔGifsy-2	This study
JH3877	ΔBTP1	This study
JH3878	ΔBTP5	This study
JH3881	ΔBTP1 ΔBTP5	This study
JH3940	ΔBTP1 ΔBTP5 ΔGifsy-1	This study
JH3942	ΔBTP1 ΔBTP5 ΔGifsy-1 ΔGifsy-2	This study
JH3949	ΔΦ (ΔBTP1 ΔBTP5 ΔGifsy-1 ΔGifsy-2 ΔST64B)	This study
JH3983	ΔGifsy-1, *aph-galE496*; Km^R^	This study
JH3984	ΔGifsy-2, *aph-galE496*; Km^R^	This study
JH3986	ΔST64B	This study
JH3987	P_dinI-gfoA_ from 14028s (C→T substitution, position 2,790,162 in D23580 chromosome)	This study
D23580 Δ*recA*	D23580 with *recA* gene replaced with *nptII* from pKD13 and subsequently flipped out using Flp recombinase encoding pCP20 plasmid	This study
**14028s derivatives**		
MA5958	14028s, WT	L. Bossi
MA6684	ΔGifsy-1 ΔGifsy-2 *bio-106*::Tn*10 galE496*; Tc^R^	L. Bossi, Campoy et al., 2006
**LT2 derivatives**		
LT2	LT2, WT	Zinder and Lederberg, 1952
MA8508	Gifsy-1[-] Gifsy-2[-] Fels-2[-] Fels-1[Δ(*int*-attR)*104::cat*]	L. Bossi, Lemire et al., 2011
LT2 [BTP1]	LT2 derivative MA8508 lysogenised with phage BTP1	This study
LT2 [P22]	LT2 derivative MA8508 lysogenised with phage P22 (wildtype)	This study
LT2 Δ*rec*	LT2 derivative MA8508 with *recA* gene replaced with *nptII* from pKD13 and subsequently flipped out using Flp recombinase encoding pCP20 plasmid	This study
**4/74 derivative**		
4/74	4/74, WT	Rankin and Taylor, 1966
***E. coli***		
S17-1 λ*pir*	*pro thi hsdR recA* chromosome::RP4-2 Tc::Mu Km::Tn*7*/λ*pir*; Tp^R^, Sm^R^	Simon et al., 1983
**Plasmids:**		
pEMG	Suicide plasmid; Km^R^	Martínez-García and de Lorenzo, 2011
pKD13	*nptII*-cassette template plasmid; Km^R^	Datsenko and Wanner, 2000
pKD4	*aph*-cassette template plasmid; Km^R^	Datsenko and Wanner, 2000
pKD4-I-*Sce*I	pKD4 derivative with an I-*Sce*I site cloned upstream of *aph*; Km^R^	This study
pKD46	λ Red recombination plasmid, arabinose-inducible; Ap^R^	Datsenko and Wanner, 2000
pKD46 *bla*::Tn*10*	pKD46 derivative; OxyTc^R^	Passaris et al., 2014
pCP20	Plasmid carrying the Flp recombinase to remove kanamycin resistance from pKD13 derived resistance cassette insertions; Ap^R^	Cherepanov and Wackernagel, 1995
pCP20-TcR	Derivative of plasmid carrying the Flp recombinase to remove kanamycin resistance from pKD4 derived resistance cassette insertions; Tc^R^	Kintz et al., 2015
pNAW15	Suicide plasmid pEMG::*attB*^Gifsy-1^; Km^R^	This study
pNAW16	Suicide plasmid pEMG::*attB*^Gifsy-2^; Km^R^	This study
pNAW17	Suicide plasmid pEMG::*attB*^BTP1^; Km^R^	This study
pNAW18	Suicide plasmid pEMG::*attB*^BTP5^; Km^R^	This study
pNAW42	Suicide plasmid pEMG::*attB*^ST64B^; Km^R^	This study
pSIM5-*tet*	λ Red recombination plasmid, temperature-inducible; Tc^R^	Koskiniemi et al., 2011
pSW-2	Plasmid for *m*-toluate-inducible expression of the I-SceI enzyme; Gm^R^	Martínez-García and de Lorenzo, 2011

*^a^Relevant antibiotic resistances are indicated by ^R^: Ap, ampicillin; Gm, gentamicin; Km, kanamycin, OxyTc, oxytetracycline; Sm, streptomycin; Tc, tetracycline; Tp, trimethoprim. For the purpose of clarity the natural resistances of D23580 and its derivatives are not indicated.*

### Phage Enumeration and Plaquing

Phage enumeration and plaque isolation were carried out using the double layer agar technique. One milliliter of bacterial culture containing phage was filtered through a 0.22 μm filter to remove bacterial cells, and diluted appropriately in sterile LB. Dilutions were applied in 10 μl drops to 4 ml 0.4% LB agar lawns seeded with 100 μl indicator strain (∼10^8^ CFU) and incubated overnight at 37°C.

### Genetic Techniques

All oligonucleotide primers used in this study are listed in Supplementary Table S1. For cloning and recombineering procedures, PCR reactions were performed with Q5 High-Fidelity DNA polymerase (New England Biolabs M0491S) according to the manufacturer’s instructions. The PCR fragments obtained from the constructed strains and the DNA inserts cloned into plasmids were sequenced by GATC Biotech (Konstanz, Germany). Recombineering procedures were carried out using the λ Red recombination plasmids pSIM5-*tet* and pKD46 in D23580 and 14028s/LT2 backgrounds, respectively (Datsenko and Wanner, 2000; Koskiniemi et al., 2011). Electro-competent cells of *Salmonella* carrying pSIM5-*tet* or pKD46 were prepared and transformed with the indicated PCR fragment as previously reported (Yu et al., 2003; Datta et al., 2006; Lemire et al., 2011) and recombinants were selected on LB agar plates supplemented with antibiotic. Generalized transduction was performed with the highly efficient transducing phage P22 HT 105/1 *int-*201 (Schmieger, 1972). As D23580 is highly resistant to P22 infection (Kintz et al., 2015), high numbers of P22 transducing particles were necessary to obtain transductants. Therefore, 200 μl of stationary phase culture of the recipient strains were mixed with 100 μl of the P22 lysates. After 1 h of incubation at 37°C, transductants were selected on LB-Km agar plates. Transductants were grown to stationary phase in LB containing 10 mM EGTA (Sigma-Aldrich, E3889) and Km. Cultures were streaked on indicator Green Plates and P22-free colonies were selected for further experiments (Maloy, 1990).

### Scarless Prophage Curing

The prophages of *S.* Typhimurium D23580 were cured according to the scarless genome editing technique developed in the de Lorenzo Lab (Martínez-García and de Lorenzo, 2011). The phage attachment site (*attB*) of Gifsy-1^D23580^, Gifsy-2^D23580^, BTP1, BTP5, and ST64B^D23580^ were PCR amplified from *S.* Typhimurium prophage-cured strains or naturally naive strains with the appropriate primers and cloned into the suicide plasmid pEMG, as summarized in Supplementary Table S2. *Escherichia coli* S17-1 λ*pir* was used as host strain for the resulting suicide plasmids pNAW15 (*attB*^Gifsy-1^), pNAW16 (*attB*^Gifsy-2^), pNAW17 (*attB*^BTP1^), pNAW18 (*attB*^BTP5^) and pNAW42 (*attB*^ST64B^). The suicide plasmids were mobilized from S17-1 λ*pir* into the recipient *S.* Typhimurium strains by conjugation. The donor S17-1 λ*pir* strains harboring the pEMG derivatives and the *Salmonella* recipient strains were grown overnight in LB-Km and LB, respectively. Cells were washed once with LB, 500 μl of both cell suspensions were mixed and bacteria were concentrated in 40 μl of LB. The mixtures were dropped on LB agar plates and incubated for mating for 6 h at 37°C. Bacteria were suspended in LB and *Salmonella* transconjugants having integrated the pEMG derivative plasmids by homologous recombination were selected on LB agar containing Km and Cm. The inserted pEMG encompasses two I*-Sce*I restriction sites that are used as counter-selective markers in the presence of the I-SceI restriction enzyme. Therefore, transconjugants were transformed with the pSW-2 plasmid and the transformants were selected on LB agar containing Gm and 1 mM *m-*toluate (prepared with *m*-toluic acid, Sigma-Aldrich T36609 and NaOH to pH 7.0), stimulating the production of the I-SceI enzyme from pSW-2 (Martínez-García and de Lorenzo, 2011). To optimize the transformation yield, pSW-2 was purified from D23580/pSW-2. The transformants having lost the integrated pEMG by a second homologous recombination were identified by their Km sensitive phenotype (Km^S^). The excision of the prophages was assessed in Km^S^ clones by PCR using the primers used to amplify the *attB* of the corresponding prophages (Supplementary Table S2). Finally, the unstable pSW-2 was eliminated from the clones of interest by two passages in LB.

### Construction of Mutant *Salmonella* by λ Red Recombineering

The *galE496* allele (premature stop codon TAG on the 326^th^ codon of *galE*) of MA6684 was transferred into D23580 derivative strains by a two-steps procedure. An *aph* (Km^R^) cassette was amplified from pKD4 with primers NW_86 and NW_87 and was inserted by λ Red recombination into the intergenic region *STM0777-galE496* of MA6684. Due to the defective O-antigen of the *galE* mutants, the resulting strain was grown in LB supplemented with 0.2% of glucose and 0.02% of galactose for the preparation of a P22 lysate (Butela and Lawrence, 2012). The *aph*-*galE496* module was subsequently transduced into D23580 derivative strains and transductants were screened by PCR with the primers NW_61 and NW_82 and checked by sequencing to confirm the presence of *galE496*. The functional promoter of the antirepressor gene (*gfoA*) in Gifsy-1 of *S.* Typhimurium 14028s was inserted in place of the corresponding promoter of D23580 (Lemire et al., 2011). Therefore, a single nucleotide substitution (C→T position 2790162) was introduced into the promoter controlling *dinI*(*STMMW_26401*)-*gfoA(STMMW_26391)* transcription (P*_dinI_*_-_*_gfoA_*) of D23580 by a protocol based on the principle described by Blank et al. (2011). Due to the MDR phenotype of D23580, a plasmid harboring an I-*Sce*I-*aph* module was first constructed: the I-*Sce*I restriction site was inserted upstream of *aph* in pKD4, using the Site-Directed Mutagenesis method with primers NW_161 and NW_162 (Laible and Boonrod, 2009). The resulting plasmid (pKD4-I*-Sce*I) was used as template to insert an I-*Sce*I-*aph* module into P*_dinI_*_-_*_gfoA_* by λ Red recombination, using primers NW_214 and NW_215. This module was transduced into a clean D23580 background and the resulting strain, D23580 I-*Sce*I-*aph-dinI-gfoA*, was transformed with pSIM5-*tet* for a second λ Red recombination. The functional P*_dinI_*_-_*_gfoA_* of *S.* Typhimurium 14028s was PCR-amplified using the primers NW_194 and NW_195 and the resulting fragment (319 bp) was electroporated into D23580 I-*Sce*I-*aph-dinI-gfoA* carrying pSIM5-*tet*. To select the recombinants, new electro-competent cells were directly prepared after the electroporation, and bacteria were transformed with pSW-2. After selection on LB agar containing Gm and *m-*toluate, colonies were screened by PCR with primers NW_196 and NW_197 and the resulting PCR fragments were sequenced to confirm the mutation. Two passages in LB were used to eliminate pSW-2. To construct a RecA^-^ LT2 strain (LT2 derivative MA8508 was used, **Table 1**), the *aph* cassette was first amplified from pKD13 with primers RecA_pKD13_Fw and RecA_pKD13_Rev, and then inserted by λ Red recombination into MA8508 pKD46. Kanamycin resistance was subsequently removed using the Flp recombinase encoded in the pCP20 plasmid. The same methodology was applied to D23580 to obtain the D23580 RecA^-^ strain, but using the pKD46 *bla*::Tn*10* and pCP20-TcR plasmids instead, as ampicillin resistance in D23580 (Kingsley et al., 2009) prohibits the use of the original pKD46 and pCP20 plasmids.

### Isolation of BTP1 Lysogens

Exponential phase cultures of susceptible host strains and BTP1 phage were mixed and plated using the double layer agar technique as described above. BTP1 containing plaques were identified using primers for the *pid* gene, pid_Fw, and pid_Rev (Supplementary Table S1). Plaques that were positive for *pid* were further picked and purified on Green indicator plates (Maloy, 1990). This medium contains glucose as a carbon source, and a pH indicator dye that turns dark green at sites where phage infection causes cell lysis and the concomitant release of organic acids, while the lysogens remain pale green. Candidate LT2 [BTP1] lysogens were picked onto fresh Green Plate and cross streaked against BTP1 phage to confirm resistance of the lysogens to superinfection by BTP1.

### Determination of Prophage Viability

Viable phages were detected in the supernatant of D23580 WT after 16 h culture. Where chemical induction was required, mitomycin C (Sigma-Aldrich M4287) was added to exponentially growing cultures (OD_600_ = 0.3) at a final concentration of 2 μg/ml. Phages were enumerated using the double layer agar technique as described above using specific indicator strains to detect each phage (**Table 2**). Prophage viability was defined as the ability to form infective phage particles with or without chemical induction.

**Table 2:** Detection of active prophage in D23580 by plaque formation on specific indicator strains for each D23580 prophage.

		Specific phage titer (PFU/mL) in D23580 WT supernatant	
Specific indicator strain	Target prophage	Non-induced	+ Mitomycin C
D23580 ΔBTP1	BTP1	1 × 10^9^	2 × 10^10^
D23580 ΔGifsy-2 *galE496*	Gifsy-2 (BTP2)	Not detected	Not detected
D23580 ΔST64B	ST64B (BTP3)	Not detected	Not detected
D23580 ΔGifsy-1 *galE496*	Gifsy-1 (BTP4)	Not detected	Not detected
D23580 ΔBTP5	BTP5	Not detected	Not detected
D23580 ΔGifsy-1 *galE496*	Gifsy-1 P*_dinI-gfoA_*^14028s^	1 × 10^4^	2 × 10^6^
D23580 WT	Putative superimmune phage (control)	Not detected	Not detected

		**Specific phage titer (PFU/mL) in D23580 ΔBTP1 supernatant**	

D23580 ΔΦ (All five prophages deleted)	Gifsy-1, Gifsy-2, ST64B, or BTP5	Not detected	Not detected

*‘Not detected’ indicates spontaneous phage induction at less than the detection threshold of 1 × 10^2^ PFU/ml. D23580 was included as an indicator strain to confirm that the strain does not support plaques from induced phages present its own supernatant. To control for the possibility that other prophages may be super excluding the target phage in the specific indicator strains, supernatant from D23580 ΔBTP1 was plated on an indicator strain with all five prophages removed (D23580 ΔΦ), yielding no plaques.*

### BTP1 Burst Size and Spontaneously Induced Population Estimation

Burst size determination was achieved using the single step growth curve protocol previously described (Hyman and Abedon, 2009). Briefly, 50 μl of exponentially growing D23580 ΔBTP1 cells (OD_600_ = 0.3) at a density of 10^8^ CFU/ml were mixed with 50 μl BTP1 phage at a density of 10^7^ PFU/ml to achieve a multiplicity of infection (M.O.I.) of ∼0.1. After 5 min adsorption time at 37°C, 1 μl of the cell-phage mixture was diluted 1000X by addition of 999 μl sterile LB, in order to prevent further adsorption. Free (non-adsorbed) phages were enumerated by plaque assay on a susceptible indicator strain as described above. Number of infected cells was calculated as: [(number of cells x M.O.I.) – number of unabsorbed phage]. Subsequently, the number of free phage was enumerated by sampling and filtration every 10 min. Difference between the phage titer pre- and post-burst divided by the number of infected cells was determined as the burst size. Duration of time between infection and burst was determined as the latent period.

The percentage of the population undergoing spontaneous induction was estimated as [(100/average number of cells in 16 h culture)/(average number of phage in 16 h culture/burst size)].

### BTP1 Genomic DNA Isolation and Sequencing

A pure BTP1 phage stock was produced by picking and resuspending a BTP1 plaque in sterile LB and plating on indicator strain D23580 ΔΦ (JH3949). This process was repeated three times to ensure purity. A high titer BTP1 lysate (∼10^11^) was produced by mixing 10 μl of BTP1 plaque suspension with 5 ml of exponential phase (OD_600_ = 0.3) D23580 ΔΦ. BTP1 virions were concentrated by PEG precipitation. Briefly, 1 ml of PEG-8000 20% NaCl 2.5 M was added to 4 ml BTP1 lysate and incubated on ice for 2 h. Subsequently the mixture was centrifuged at 15,000 *g* for 1 h at 4°C and liquid decanted. The pellet was resuspended in 50 μl molecular grade water and treated with DNase I for 1 h at 37°C to remove any contaminating bacterial DNA. Subsequently the sample was heated to 90°C for 10 min to simultaneously deactivate DNase I and release BTP1 DNA from capsids. BTP1 DNA was purified using the Zymo Research Quick-DNA^TM^ Universal Kit (cat# D4069) as *per* the Biological Fluids and Cells protocol and eluted in 15 μl molecular grade water. Sequencing libraries were prepared using the Illumina Nextera XT DNA sample kit *per* the manufacturer’s protocol (Illumina, USA) with 1 ng of input DNA. Sequencing was performed on an Illumina MiSeq instrument using V2 chemistry (2 × 250 bp). Sequencing reads were quality trimmed using Sickle (Joshi and Fass, 2011) and assembled using Spades (Bankevich et al., 2012). The assembly was manually reordered to begin at the small terminase. Functional annotation was transferred from the D23580 (FN424405) prophage BTP1 sequence using prokka 1.11 (Seemann, 2014). Read data, assembly and functional annotation were deposited at the DDBJ/EMBL/GenBank database under the sample accession LT714109^1^ and study accession PRJEB18919.

### Bioinformatic Analyses

Prophage locations in the D23580 genome were extracted from the Genbank annotation (Accession number: FN424405) confirmed by submitting the D23580 genome sequence to PHAST (Zhou et al., 2011) and for BTP1 and BTP5, precise prophage boundaries were determined by analysis of the *att* sites. The genome of *S.* Typhimurium strain 4/74 (Accession number: CP002487) was used as a comparator to identify D23580-specific prophages. Genome comparison was done using BLASTn alignment with default parameters and visualized using the Artemis Comparison Tool (ACT; Carver et al., 2008). Prophage ORFs were characterized by uploading individual prophage sequences to the RAST server, and visualized using the SEED viewer in the context of the closest phage relatives in the RAST database (Aziz et al., 2008; Overbeek et al., 2014). Protein functions were inferred based on homologies to known proteins by the BLASTp and psiBLAST server of NCBI using the UniProt/Swiss-Prot database. Morphological classification was made based on similarity to other well-studied phages. Clustering of functional gene groups was based on a similar analysis by Zou et al. (2010). Assessment of the conservation of the functionally degradative mutations identified in Gifsy-1^D23580^, ST64B^D23580^, and Gifsy-1^D23580^ across other ST313 isolates was achieved using BLASTn. Genome assemblies were downloaded from Enterobase^2^.

### Transmission Electron Microscopy (TEM)

To prepare samples for Transmission Electron Microscopy (TEM), 10 μl of the supernatant of a 16 h D23580 culture was pipetted onto a carbon/Pioloform-coated copper 200-mesh grid, and allowed to adhere for 10 min. To image phage infections, the supernatant of a 16 h D23580 culture containing virions was added to mid-exponential phase *S.* Typhimurium 4/74 (OD_600_ = 0.3) cells in LB media, and incubated at 37°C for 20 min before TEM sample preparation. The grids were subsequently washed twice in distilled water for 2 min, negative stained with 2% uranyl acetate for 1 min and examined using a FEI 120 kV Tecnai G2 Spirit BioTWIN transmission electron microscope at the EM Unit, University of Liverpool.

## Results

### The Prophage Repertoire of D23580

**FIGURE 1:**
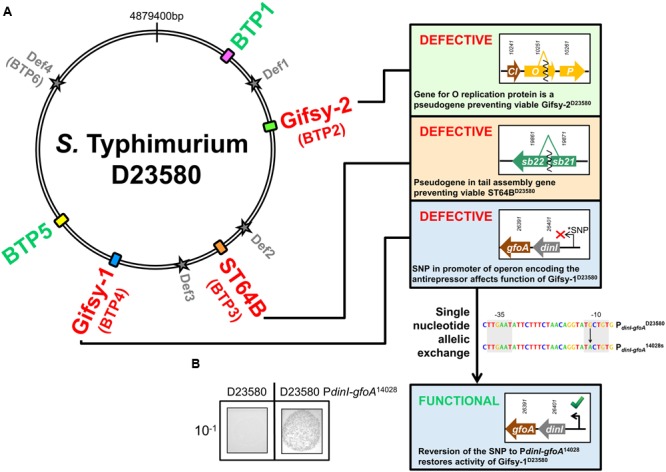
**Prophage map of ST313 *Salmonella* Typhimurium and determination of phage viability. (A)** *S.* Typhimurium ST313 representative strain D23580 contains 5 full-length prophage sequences and four prophage remnants (Def1-4). BTP1 and BTP5 are novel phages that have not been described in other sequenced *Salmonella.* Panels show important phage proteins for replication and tail gene assembly are pseudogenes in D23580, making the Gifsy-2^D23580^, ST64B^D23580^, and Gifsy-1^D23580^ prophages non-viable (numbers above arrows representing genes are D23580 identifiers *STMMW*_). Gifsy-1^D23580^ is shown to be non-viable due to a SNP in the promoter sequence of the operon encoding the GfoA antirepressor. **(B)** Restoration of the promoter results in viable phage production. The nucleotide sequences of the promoters P*_dinI-gfoA_*^D23580^ and P*_dinI-gfoA_*^14028s^ are represented and the black arrow indicates the nucleotide that was exchanged to restore Gifsy-1^D23580^ function. 10^-1^ indicates that the culture supernatant used in the spot assay represented was diluted 1:10.

Six prophages were originally annotated in the genome of ST313 representative strain D23580 (Kingsley et al., 2009). In fact, D23580 contains a total of nine phage-derived sequence islands (**Figure 1A**; **Table 3**). Four short prophage-remnants were named Def1-4, using the nomenclature defined for *S.* Typhimurium strain LT2 (Casjens, 2011). Def1-4 are found in all Typhimurium complete genomes currently available on NCBI (accessed 28th July 2016) and are likely to be ancestral to the serovar (Casjens, 2011). One feature designated ‘BTP6’ was included in the original annotation of D23580 (accession FN424405; Kingsley et al., 2009) and is located at the position of prophage remnant Def4. The remaining five prophage regions, designated BTP1-5, are full-length prophages (**Table 3**) that contain all the functional gene clusters required for phage function such as integration/excision, immunity, lysis, capsid and tail gene clusters. Three represent well-characterized prophages, commonly found in *S.* Typhimurium genomes: BTP2 is Gifsy-2^D23580^, BTP3 is ST64B^D23580^ and BTP4 is Gifsy-1^D23580^ (**Figure 1A**) (Figueroa-Bossi et al., 2001; Tucker and Heuzenroeder, 2004). BTP1 and BTP5, however, are novel prophages that are only found in ST313 and not present in the 187 complete *Salmonella* genomes available in the NCBI complete genome database (accessed 28th July 2016). Together, the phage-derived sequence islands harbor many characterized virulence-related genes in *S.* Typhimurium including genes encoding effector proteins and O-antigen modification genes (*gtr* genes) (**Table 3**).

**Table 3:** Location of all prophage or remnant prophage elements in the D23580 chromosome and genes related to *Salmonella* virulence encoded on each element.

Prophage/remnant	Location on D23580 chromosome	Length (bp)	Virulence-related genes
**BTP1**	368797 – 409321	40,525	***st313-td*** (*STMMW_03531*), ***gtrCc*** (*STMMW_03911*), ***gtrAc*** (*SMMTW_03921*)
**Def1** (SPI-16)	652441 – 656532	4,091	***gtrBa*** (*STMMW_06241*), ***gtrAa*** (*STMMW_06251*)
**Gifsy-2^D23580^** (BTP2)	1094166 – 1140420	46,254	***gtgA*** (*STMMW_10381*), ***sodCI*** (*STMMW_10551*), ***sseI*** (*STMMW_10631-*pseudogene), ***gtgE*** (*STMMW_10681*) ***gtgF*** (*STMMW_10691*)
**Def2**	1945242 – 1951433	6,191	***sopE2*** (*STMMW_18441*)
**ST64B^D23580^** (BTP3)	2065743 – 2105830	40,087	***sseK3*** (*STMMW_19812*)
**Def3** (inc. SPI-12)	2354483 – 2369638	15,155	***sspH2*** (*STMMW_22721*)
**Gifsy-1^D23580^** (BTP4)	2753352 – 2803478	50,126	***gogB*** (*STMMW_26001*), ***gipA*** (*STMMW_26191*), ***gtgA*** (*STMMW_26331*),
**BTP5**	3370278 – 3401458	31,181	–
**Def4**	4440630 – 4461045	20,415	***gtrBb*** (*STMMW_41541*), ***gtrAb*** (*STMMW_41551*)

*All elements are present in ST19 representative strain 4/74, apart from BTP1 and BTP5.*

### Novel Prophage BTP1

The gene content and synteny of BTP1 resembles P22, suggesting that BTP1 belongs to the *Podoviridae* family of short-tailed phages (Ackermann, 2009). Functional annotation of BTP1 genes identified a number of putative cargo loci on the prophage: independent transcriptional units that are not required for the prophage lytic or lysogenic cycles (Cumby et al., 2012). Like P22, BTP1 contains a *gtrAC* operon (*STMMW_03911-03921*) (Supplementary Figure S1A) which encodes a glycosyltransferase enzyme that modifies the O-antigen of the bacterial lipopolysaccharide (Kintz et al., 2015). This glycosyltransferase mediates the acetylation of the rhamnose residue of the O-antigen and is classified as a family 2 GtrC protein, which are typically associated with invasive *Salmonella* serovars such as Typhi and Paratyphi A (Kintz et al., 2015). Previously it has been shown that a BTP1 lysogen is highly immune to superinfection by P22, suggesting the two phages share a superinfection immunity system, likely mediated by the *gtr* glycosyltransferase. The *st313-td* gene (*STMMW_03531*) is encoded immediately downstream of the CI repressor locus, and its deletion decreases survival within murine macrophages, and results in attenuation in a mouse infection model (Herrero-Fresno et al., 2014). At the time of description of the *st313-td* gene, prophage BTP1 had not been characterized and therefore the fact that *st313-td* is BTP1-encoded has not been previously explored. Additionally, the *pid* ORFan locus (*STMMW_03751*) is present in BTP1. The P22-encoded Pid regulatory protein de-represses the host *dgo* operon in *S.* Typhimurium LT2 (Cenens et al., 2013). The complete functionally annotated map of BTP1 is shown in Supplementary Figure S1A. The chromosomal context of the BTP1 *attB* is shown in Supplementary Figure S2A, and the core *att* sequence for BTP1, is given in Supplementary Table S3.

**FIGURE 2:**
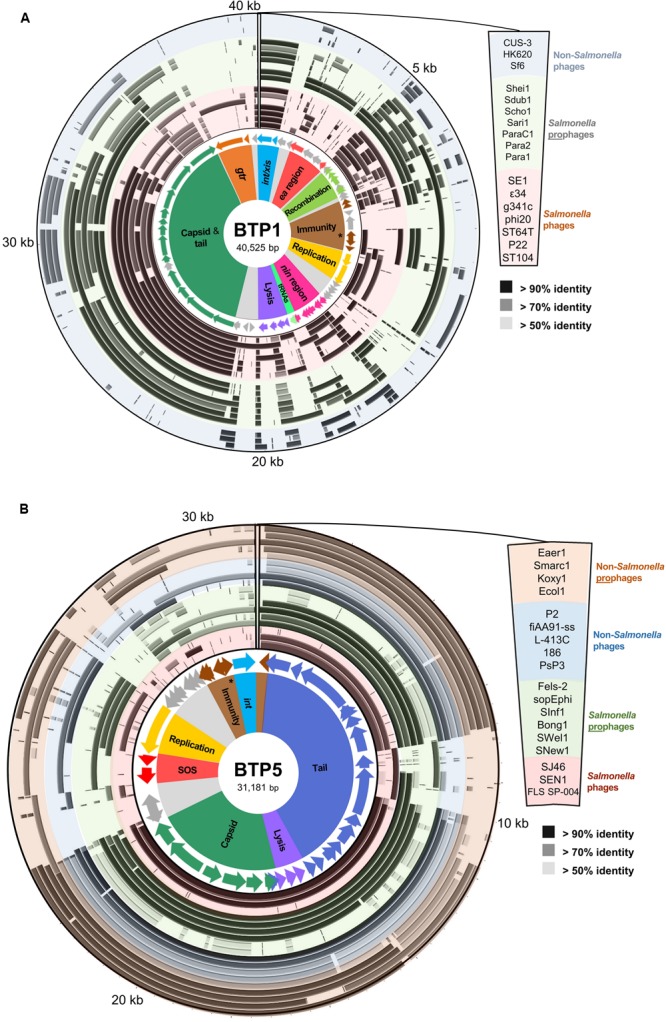
**Functional genetic architecture of BTP1 and BTP5 and homology to sequenced *Salmonella* phages and prophages, and non-*Salmonella* phages and prophages.** Starting from the inside, circular panels represent functional clusters of genes: ORFs colored according to functional cluster with functionally unknown ORFs colored gray; BLAST homology to other *Salmonella* phage sequences (red-shaded ring), *Salmonella* prophage sequences (green-shaded ring), non-*Salmonella* phages (blue-shaded ring) and non-*Salmonella* prophages. The order of sequences represented by each concentric ring of the BLAST homology panel are shown on the right. The BTP1 chromosome is shown in panel **(A)**, and the BTP5 chromosome is shown in panel **(B)**. Details of the sequences used in this analysis can be found in the Supplementary Tables S4 and S5. The repressor gene of each prophage is indicated with an asterisk in the respective immunity regions.

A comparative genomic analysis assigned putative function to 43/64 (62.5%) of the annotated open reading frames (ORFs) of BTP1. A comparison with model phages P22 and Lambda revealed that BTP1 ORFs can be classified into 10 functional blocks (**Figure 2A**, full details of the sequences used in this analysis can be found in Supplementary Table S4). The functional blocks show mosaic conservation in other phage and prophage genomes suggesting functional overlap and recombination. Similarity to other phages extends beyond phages infecting the *Salmonella* genus, as BTP1 shares sequence identity with P22-like phages of *E. coli* and *Shigella* (CUS-3, HK620, and Sf6). Some BTP1 functional blocks are rarer among the sequences sampled in this analysis than others. For example, the BTP1 *gtr* block is only found in *Salmonella* phage 𝜀34. Others, such as the capsid and tail block, *int/xis* block and recombination block, are found in the majority of sampled sequences. We found that the immunity regions of the P22-like phages and prophages also show mosaicism, with some phages sharing an almost identical immunity region, and other phages completely lacking homology to the immunity region of BTP1. For example, there is no significant nucleotide homology between the immunity regions of BTP1 and P22. In contrast, prophage ST104 contains a very similar immunity region to BTP1 (Supplementary Figure S3). The differences between the immunity regions suggest the regulation of lysogeny may differ between these prophages. We note that the *sieB* gene, encoding an asRNA-based superinfection exclusion system (Ranade and Poteete, 1993), is present in the immunity region of P22 and ST104 and is absent from the immunity region of BTP1.

### Novel Prophage BTP5

The BTP5 structural genes suggest it is a member of the *Myoviridae* family of long contractile-tailed phages and it shares sequence similarity with P2-like phages including P2, WΦ, and 186 which infect *E. coli*. P2 has been described as the prototype for the non-inducible class of temperate phages, as its repressor protein lacks an identified RecA cleavage site (Kalionis et al., 1986), and P2-like phages are known to be associated with satellite phages (Calendar, 2006). However, BTP5 contains two genes that show homology to the SOS operon of coliphage 186 (Brumby et al., 1996): *tum* (*STMMW_32041*) and *orf97* (*STMMW_32031*), which have been shown to function as an antirepressor system and facilitate induction of coliphage 186 (Shearwin et al., 1998). No function was able to be inferred based on sequence homology to other studied phages for 7/42 of BTP5 ORFS. The complete functionally annotated map of BTP5 is shown in Supplementary Figure S1B. The chromosomal context of the BTP5 *attB* is shown in Supplementary Figure S2B, and the core *att* sequence for BTP5 is given in Supplementary Table S3.

Comparison of BTP5 to other P2-like phages and prophages suggests that there is less genetic mosaicism than in the P22-like group (**Figure 2B**, full details of the sequences used in this analysis are given in Supplementary Table S5). The majority of the P2-like phages and prophages show conservation of nearly the entire capsid and tail gene clusters, which make up ∼60% of the phage chromosome. Structural gene homology is not restricted to the genus *Salmonella*, and BTP5 shares >70% sequence identity to the structural genes of phages and prophages from other Enterobacteriaceae including *Escherichia, Klebsiella, Serratia*, and *Enterobacter*. However, outside of the structural gene clusters, there is limited homology to other phages or prophages, suggesting that BTP5 may represent novel phage biology.

### Testing Prophage Viability with Specific Indicator Strains

Prophages were defined as viable if functional (infectious) phage particles could be produced, with or without induction. In order to test viability of each of the D23580 prophages, we created a series of naive indicator strains of D23580 using scarless genome editing to delete each prophage (see Materials and Methods). This resulted in a collection of five isogenic D23580 derivative strains, each lacking a single prophage (**Table 2**) and therefore harboring intact *attB*. As Gifsy phages have previously been shown to yield visible plaques only on hosts with short LPS, due to the receptor OmpC being blocked by the O-antigen (Ho and Slauch, 2001), an additional *galE496* mutation was introduced to facilitate detection of Gifsy-phage plaques. Specific indicator strains used to detect each phage are shown in **Table 2**.

### BTP1 Represents a Novel Species of the Genus *P22virus*

The supernatant from a 16 h LB culture of strain D23580 yielded circular plaques of ∼1 mm in diameter with turbid centers on the specific indicator strain D23580 ΔBTP1 at an unusually high abundance of ∼10^9^ PFU/ml (plaque-forming units *per* ml). Mitomycin C induction increased the phage titer 10-fold to ∼10^10^ PFU/ml (**Table 2**).

We propose that BTP1 represents a new species designated “Salmonella virus BTP1” within the *P22virus* genus. This is consistent with the International Committee on Taxonomy of Viruses (ICTV) criterion that members of a proposed species differ from those of other species by more than 5% at the DNA level as confirmed with the BLASTN algorithm. The BTP1 phage genome was sequenced and re-ordered to begin at the small terminase subunit, to conform to the reference sequence for the *P22virus* genus, *Salmonella virus P22* (accession: BK000582). No sequence differences were found between the BTP1 prophage and phage sequences. The annotated genome and sequencing reads for the BTP1 phage have been deposited at the DDBJ/EMBL/GenBank database under the accession LT714109^3^ and study accession PRJEB18919. The proposed species “Salmonella virus BTP1” has been assigned the taxon ID 1934252.

### Novel Prophage BTP5 May Not Be Viable

The supernatant from a 16 h LB culture of strain D23580 did not form plaques on the specific indicator strain D23580 ΔBTP5, even after mitomycin C induction. Therefore, due to the inability to observe plaques, and lack of obvious functional degradation of the BTP5 prophage sequence, we were unable to verify the viability of prophage BTP5.

### Prophages Gifsy-2, ST64B, and Gifsy-1 are Defective in ST313 *S*. Typhimurium

Supernatant from non-induced and mitomycin C-treated D23580 16 h cultures did not form plaques on specific indicator strains for Gifsy-2^D23580^, ST64B^D23580^, and Gifsy-1^D23580^ (D23580 ΔGifsy-2^D23580^ *galE496*, D23580 ΔST64B^D23580^, and D23580 ΔGifsy-1^D23580^ *galE496*, respectively) (**Table 2**). Closer inspection of the genome revealed evidence for functional inactivation of Gifsy-2^D23580^, Gifsy-1^D23580^, and ST64B^D23580^ by mutation.

All coding genes remain intact in Gifsy-1^D23580^ compared to Gifsy-1^14028^, where Gifsy-1 is known to be functional (Figueroa-Bossi and Bossi, 1999), with no evidence for functional degradation *via* pseudogene formation. However, a single nucleotide polymorphism (SNP) was detected in the -10 promoter region of the *dinI* gene of Gifsy-1^D23580^ compared to the same region in *S.* Typhimurium 14028s (Gifsy-1^14028s^) (Bossi et al., 2003). It has previously been shown that the anti-repressor gene of Gifsy-1, *gfoA*, is located immediately downstream of *dinI* and is co-transcribed from the *dinI* promoter (P*_dinI-gfoA_*), making *gfoA* essential for the induction of Gifsy-1 phage (Lemire et al., 2011). This finding prompted us to test if the promoter SNP is responsible for the lack of detectable Gifsy-1 in the D23580 supernatant (**Table 2**). Single nucleotide allelic exchange of P*_dinI-gfoA_*^D23580^ with P*_dinI-gfoA_*^14028s^ yielded viable (infectious) Gifsy-1 viruses that formed plaques on D23580 ΔGifsy-1. D23580 P*_dinI-gfoA_*^14028s^ produced a Gifsy-1 titer consistent with that reported for 14028s, both with and without mitomycin C induction (Figueroa-Bossi and Bossi, 2004) (**Figure 1B**). The fact that a single modified nucleotide caused the production of functional phage shows that the P*_dinI-gfoA_* SNP is responsible for lack of Gifsy-1 viability in D23580.

ST64B is defective in *S.* Typhimurium 14028s due to a frame-shift mutation in a tail assembly gene that prevented assembly of viable phage particles, and created two short gene fragments annotated *sb21* and *sb22* (Figueroa-Bossi and Bossi, 2004). The tail gene frame-shift mutation is conserved in ST64B^D23580^ (D23580 genes *STMMW_19871* and *STMMW_19861* are the homologs of *sb21* and *sb22*, respectively). Together with the failure of D23580 supernatant to produce plaques on a ST64B-sensitive host strain, we conclude that ST64B is similarly defective in *S.* Typhimurium ST313.

We noticed that the gene encoding the O replication protein of Gifsy-2^D23580^ was a pseudogene; a 71 bp deletion in the middle of the coding sequence caused a frame shift at position 1,102,739 on the D23580 chromosome, and the generation of a truncated O protein. The O protein of lambdoid phages is required for replication of the phage chromosome during lytic growth and contains the phage origin of replication (Taylor and Wegrzyn, 1995). As the replication of the phage chromosome is an essential part of the lytic cycle, we suggest that this pseudogene is likely to be responsible for the lack of viable Gifsy-2^D23580^ viruses when D23580 is grown with and without mitomycin C.

The SNPs associated with the loss of functionality of Gifsy-1^D23580^ and ST64B^D23580^ described above were conserved across 182 genomes of ST313 isolates from Malawi (representing both lineage I and lineage II of ST313) (Supplementary Table S6) suggesting that Gifsy-1 and ST64B are defective in all ST313 isolates surveyed. However, the deletion responsible for creating the *O* replication pseudogene of Gifsy-2^D23580^ was only present in lineage II strains, with ST313 lineage I isolates encoding a full-length protein. Therefore, Gifsy-2 may still be viable in ST313 lineage I.

Prophages may contain superinfection exclusion systems that can interfere with other prophages. To control for the possibility that other D23580 prophages may be excluding the target phage in the specific indicator strains, we constructed a strain with all five prophages deleted (designated D23580 ΔΦ). When supernatant from D23580 ΔBTP1 was plated onto a lawn of D23580 ΔΦ, no plaques were observed, supporting the conclusion that of the five D23580 prophages, only BTP1 is capable of plaque formation (**Table 2**).

### BTP1 Secondary Structure Prediction Based on TEM and Sequence Similarity with Structural Genes of Phage P22

**FIGURE 3:**
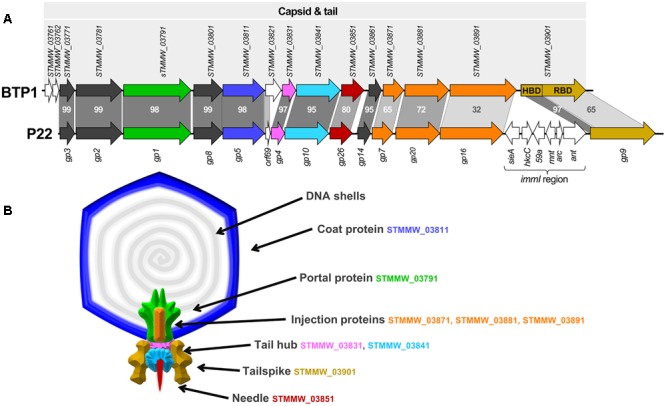
**Structural gene cluster of BTP1 and virion structural model based on phage P22. (A)** The structural gene cluster of prophage BTP1 consists of 13 ORFs of known function in virion structure and assembly (shown as colored arrows). Additionally there are three ORFs of unknown function (shown as white arrows). Model phage P22 contains an almost identical structural cluster, but with the major addition of the *imml* region. Gray bars represent percentage amino acid identity of gene homologs. Published CDS annotation is shown for both regions. The gene encoding the tailspike (*STMMW_03901*) is separated into its two functional domains: the head-binding domain (HBD) and the receptor-binding domain (RBD). **(B)** Diagram of predicted structure and location of structural proteins in BTP1, based on cryoEM reconstruction of phage P22 by Tang et al. (2011). BTP1 shares near identical structural gene content with P22 and colored protein segments correspond to the protein coding genes in **(A)**.

Comparison of the BTP1 and P22 late genes using tBLASTx shows that the structural regions share extensive homology, with the notable absence of the P22 immunity region (*imml*) in BTP1 (**Figure 3A**). Due to the high level of sequence identity and the detailed three-dimensional structural information that is available for many P22 proteins, it was possible to map the protein coding genes of BTP1 onto a structural model of P22 (**Figure 3B**). The capsid and tail genes of BTP1 show a high level of similarity to P22, with the majority of proteins sharing >90% amino acid identity. The genes involved in DNA injection (*STMMW_03871-91*) show less homology, suggesting subtle differences between the mechanism of DNA entry of the phages. The tailspike protein of P22-like phages consists of a short head-binding domain (HBD) which attaches the tailspike to the capsid, and a receptor-binding domain (RBD) responsible for recognition and binding to the bacterial receptor (Steinbacher et al., 1997). The HBD of the BTP1 tailspike shares 97% sequence identity with P22, however, the RBD is more divergent and is just 65% identical. This may indicate differences in the enzymatic activity of the tailspike protein, which in P22 is known to have endorhamnosidase activity (Iwashita and Kanegasaki, 1973).

**FIGURE 4:**
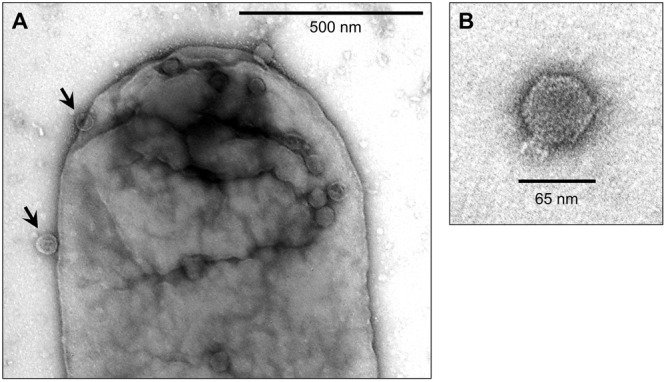
**Transmission Electron Microscopy of BTP1 virions infecting *S.* Typhimurium 4/74.** BTP1 virions from D23580 supernatant were added to mid-exponential phase 4/74 (OD_600_ = 0.3) cells in liquid culture. After 20 min samples were prepared for TEM with negative staining using uranyl acetate. **(A)** BTP1 virions adsorbing to a 4/74 cell (black arrows). **(B)** An isolated BTP1 virion.

The morphology of the BTP1 phage was determined by TEM. The purified BTP1 phage preparation contained short-tailed phages with icosahedral capsids of ∼65 nm in diameter (**Figure 4B**). The addition of purified BTP1 phages to susceptible *S.* Typhimurium 4/74 cells allowed the same short-tailed phages adsorbing to the surface of the bacterial cells (**Figure 4A**). Empty ‘ghost’ virions were also seen, suggesting that some phages had already adsorbed and injected DNA into the host cell at 20 min post-infection.

### Spontaneous Phage Production is 10,000-Fold Greater in a BTP1 Lysogen than a P22 Lysogen

**FIGURE 5:**
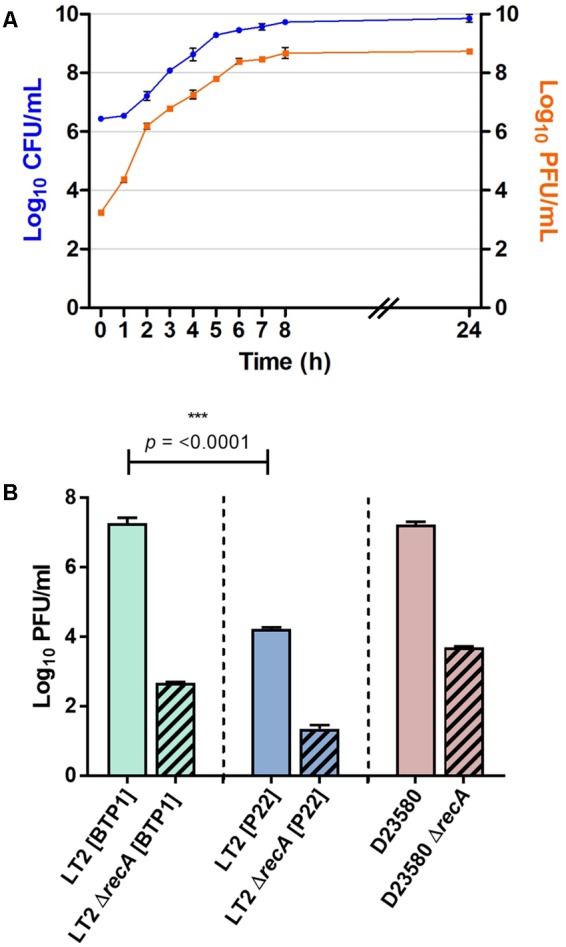
**BTP1 spontaneous induction dynamics and titer compared to phage P22. (A)** Growth curve of *S.* Typhimurium D23580 showing CFU/ml on the left *y*-axis and PFU/ml on the right *y*-axis indicating BTP1 titer. **(B)** Spontaneous induction of BTP1 and P22 in a RecA^-^ background. RecA-independent spontaneously induced phage titers are shown in strain LT2 for P22, and strains LT2 and D23580 for BTP1.

To determine whether the unusually high level of spontaneous induction (**Table 2**) is an intrinsic characteristic of the BTP1 prophage, a prophage cured derivative of *S.* Typhimurium LT2 (MA8505) was lysogenized with BTP1, and compared with LT2 lysogenized with P22. The phage titer of non-induced culture supernatants was determined. The titer of BTP1 in culture supernatants was 10,000-fold greater than the titer of P22 (**Figures 5A,B**) showing that a high frequency of spontaneous induction is an inherent characteristic of phage BTP1, independent of the D23580 host. The burst size of BTP1 in D23580 was around 600 (Supplementary Figure S4), resembling the burst size of phage P22 (Aramli and Teschke, 1999). The titer of 10^9^ PFU/ml in the D23580 non-induced 16 h culture (**Table 2**) suggests that the fraction of the cellular population undergoing phage-mediated lysis is ∼0.2%. In LT2 [BTP1], the fraction of the population undergoing phage-mediated lysis is ∼0.02%.

### There is Greater RecA-Independent Spontaneous Induction of BTP1 than P22

To determine if the high-level of BTP1 spontaneous induction was dependent on the SOS-response, we measured the spontaneously induced phage titer in a RecA^-^ BTP1 lysogen, using a RecA^-^ P22 lysogen as a comparator (**Figure 5B**). In host strain LT2 (MA8505), P22 spontaneous induction is virtually abolished in a Δ*recA* background, and there is a 3-log reduction to the order of only 10 PFU/ml. In an LT2 (MA8505) lysogen, BTP1 spontaneous induction was also dramatically reduced in the Δ*recA* background by 5-log but remained 50-times greater than that of P22 at around 500 PFU/ml. Considering that LT2 is not the native host of BTP1, we also assessed the role of RecA on spontaneous induction of BTP1 in D23580, and found a 4-log reduction in PFU/ml in a Δ*recA* background. These results show that the majority of BTP1 spontaneous induction is RecA-dependent. However, there is still considerable RecA-independent induction of the BTP1 prophage, and we note that there is 1 log more RecA-independent induction in the native D23580 background than in an LT2 (MA8505) background.

## Discussion

The spectrum of human disease associated with *S.* Typhimurium infection in SSA is clearly different to that seen in resource-rich settings. The high prevalence of immunosuppressive illness in Africa is certainly responsible for creating a permissive niche in humans, but in addition, the isolates causing iNTS disease have been shown to be genetically distinct from *S.* Typhimurium associated with gastroenteritis in resource rich settings. This raises the possibility that there may be genetic adaptation in these strains that contributes to the burden of iNTS disease seen in Africa. In particular, African ST313 strains differ from ST19 at the level of the prophage repertoire (Kingsley et al., 2009; Okoro et al., 2015). As prophages represent a significant portion of the *Salmonella* accessory genome (Bobay et al., 2014), and are frequently associated with virulence factors in *S. enterica* (Figueroa-Bossi et al., 2001), we decided to investigate *S.* Typhimurium ST313 biology in the context of its resident prophages.

Representative ST313 strain D23580 has been shown to contain five prophages (Kingsley et al., 2009). Three of these, Gifsy-2, ST64B and Gifsy-1, are present in many *S.* Typhimurium genomes, but BTP1 and BTP5 are novel prophages. The prophage repertoire of D23580 has been shown to be highly conserved in other ST313 genomes of both described ST313 lineages (Kingsley et al., 2009; Okoro et al., 2015). The presence of novel prophages in the genome of ST313 *S.* Typhimurium is not in itself surprising, as prophages are known to be highly variable in *Salmonella* populations (Figueroa-Bossi et al., 2001; Brüssow et al., 2004). However, despite their intrinsic mobility and apparent transience, prophage carriage has also been shown to be strongly associated with *Salmonella* epidemics and the association between prophage carriage and *Salmonella* disease epidemiology is highlighted by the successful use of phage typing schemes for surveillance of *Salmonella* outbreaks since the 1970s (Rabsch, 2007). Consequently, the potential contribution to the virulence of ST313 by novel prophages BTP1 and BTP5 remains intriguing. Certainly, the presence of a number of putative accessory genes in BTP1 and BTP5 warrants further investigation. Notably, one BTP1 gene, *st313-td*, has been reported to be associated with virulence in a mouse infection model (Herrero-Fresno et al., 2014). Though no mechanism of action has been described for the virulence effect of *st313-td*, if the gene proves to be important for bloodstream invasion, its high conservation among ST313 isolates would be strong evidence that prophage BTP1 contributes to the virulence of ST313. Additionally, the high level of similarity of BTP1 to the generalized-transducing phage P22 raises interesting implications for horizontal gene transfer among BTP1-susceptible *Salmonellae.* Due to their high conservation in ST313 genomes, it is tempting to speculate that prophages are important within the ecological niche of ST313, whether through immunity to environmental phage predation, permitting colonization of environmental reservoirs in Africa or by contributing to bacterial virulence through lysogenic conversion.

As well as putative cargo virulence factors, genetic analysis revealed that the BTP1 tailspike may be unusual. Amino acid sequence comparison of the BTP1 and P22 tailspike protein receptor-binding domain (RBD) shows considerable divergence (65% amino acid percent identify), suggesting the enzymatic activity of the BTP1 tailspike may differ from other P22-like phages, where it has been shown to have endorhamnosidase activity (Iwashita and Kanegasaki, 1973). In their thorough review on the evolution of P22-like phages, Casjens and Thuman-Commike included the BTP1 prophage sequence in detailed phylogenetic analysis of capsid assembly genes (Casjens and Thuman-Commike, 2011) where it is designated ‘Typh1.’ Figure 7, S1Z4 and S1Z5 in the aforementioned review show that whilst the BTP1 tailspike head-binding domain (HBD) is very closely related to P22-like phages, the RBD falls on a deep branch amongst non-P22-like *Salmonella* phages, sharing more recent common ancestors with the RBDs of sipho- and myovirus tailspikes than P22-like phages. The significance of the unusual BTP1 tailspike RBD is unclear.

Investigation of the biology of the novel prophages in ST313 revealed that the titer of phage BTP1 in the 16 h culture supernatant is in the order of 10^9^ PFU/ml without chemical induction, approximately equal to the bacterial CFU/ml, suggesting a high frequency of spontaneous induction. The high frequency spontaneous induction was maintained in a different host strain (LT2), indicating it is an intrinsic characteristic of prophage BTP1. In order to put this into context of the phage literature, we conducted a review of previously published spontaneously induced phage titers (**Table 4**). We found that the highest reports for spontaneously induced phage titers were in the order of 10^7^ PFU/ml, for prophages ϕadh and φLC3 in *Lactobacillus gasseri* and *Lactococcus lactis*, respectively. Consequently, to the best of our knowledge, BTP1 exhibits the highest spontaneously induced phage titer of any published prophage/host system.

**Table 4:** Overview of the order of magnitude of spontaneous induced phage titers from published literature and this study.

Prophage	Resident host	PFU/ml	Reference
LES400	*Pseudomonas aeruginosa*	10^2^	Fothergill et al., 2011
LESB58	*Pseudomonas aeruginosa*	10^2^	Fothergill et al., 2011
LESB65	*Pseudomonas aeruginosa*	10^3^	Fothergill et al., 2011
SPO2	*Bacillus subtilis*	10^4^	Garro and Law, 1974
ST64B	*Salmonella* Typhimurium	10^4^	Figueroa-Bossi and Bossi, 2004
P1	*Enterococcus faecalis*	10^4.5^	Matos et al., 2013
P22	*Salmonella* Typhimurium	10^5^	This study
MuSo2	*Shewanella oneidensis*	10^5^	Gödeke et al., 2011
ϕ105	*Bacillus subtilis*	10^5^	Garro and Law, 1974
ϕMMP02	*Clostridium difficile*	10^5.5^	Meessen-Pinard et al., 2012
ϕMMP04	*Clostridium difficile*	10^5.5^	Meessen-Pinard et al., 2012
Mu	*Escherichia coli*	10^6^	Howe and Bade, 1975
SV1	*Streptococcus pneumoniae*	10^6^	Carrolo et al., 2010
ϕadh	*Lactobacillus gasseri*	10^7^	Baugher et al., 2014
φLC3	*Lactococcus lactis*	10^7^	Lunde et al., 2003
BTP1	*Salmonella* Typhimurium	10^9^	This study

Our data suggest that the high spontaneously induced BTP1 titer found in D23580 supernatant is due to the spontaneous induction of ∼0.2% of the cell population. Though it could be argued that the high spontaneous induction titer of a BTP1 lysogen is a product of the high burst size (∼600), comparison with the reported spontaneous induction rates of other prophages suggests otherwise. The induced population of lambda lysogens has been reported to be ∼1 cell in 10^5^, which equates to 0.001% (Little et al., 1999; Little and Michalowski, 2010). Approximately 0.005% of *E. coli* lysogens of Stx phage H-19b were found to be spontaneously induced *per* generation (Livny and Friedman, 2004), and spontaneous induction at the molecular level was measured in 0.01–0.008% of *Corynebacterium glutamicum* ATCC 13032 CGP3 lysogens (Nanda et al., 2014). Furthermore, as the burst size of BTP1 and P22 are comparable (∼600), and we have shown the spontaneous induction titer of P22 in an LT2-derivative lysogen to be 10,000-fold lower than BTP1, we estimate that the fraction of the LT2 [P22] population undergoing spontaneous induction is 0.00002% or ∼1 cell in 10^5^, significantly lower than the rate for BTP1 and similar to that reported for phage lambda.

The exact mechanism for the dramatic induction rate difference between BTP1 and closely related phage P22 is unclear. Typically phage induction occurs by the activation of the SOS response caused by cellular stress such as DNA damage. The SOS response triggers phage induction directly or indirectly through the activated RecA protein, which, in the model phage P22, stimulates autoproteolysis of the phage CI repressor protein leading to de-repression of phage replication functions (Ptashne, 2004). However, there are a few examples of RecA-independent prophage induction (Imamovic and Muniesa, 2012), though the exact mechanism remains unknown. As the immunity regions of P22 and BTP1 show little homology (Supplementary Figure S3), it is possible that the immunity system of BTP1 is the cause of increased susceptibility to RecA-independent induction.

Spontaneous prophage induction is a well-known phenomenon in bacteria and the presence of free phages in the cultivation media of lysogens has been reported as early as the 1950s (Lwoff, 1953). However, spontaneous induction and its potential effect on host biology has not been well documented among clinically relevant bacteria. Furthermore, the effect of spontaneous prophage induction specifically on *Salmonella* infection dynamics and virulence is not well understood. There is evidence that spontaneous phage induction directly contributes to virulence in a number of other bacterial pathogens (reviewed in Nanda et al., 2015). Phage induction causes an increase in biofilm formation by releasing extracellular DNA in organisms including *Streptococcus pneumoniae* and *Shewanella oneidensis* (Carrolo et al., 2010; Gödeke et al., 2011). More recently spontaneous prophage induction was shown to modulate *Pseudomonas aeruginosa* biofilm formation by stimulating biogenesis of membrane vesicles (Turnbull et al., 2016). However, despite published associations between spontaneous prophage induction and biofilm formation, recent publications have shown ST313 to be defective at forming the biofilm-associated RDAR (red, dry, and rough) colony morphology and surface-associated biofilms (Ramachandran et al., 2016; Singletary et al., 2016).

It has also been reported that phage-mediated lysis may act as a crude method of toxin delivery, facilitating human gingival fibroblast invasion by *Aggregatibacter actinomycetemcomitans* (Stevens et al., 2013), and enabling Stx toxin release in Shiga toxin-producing *E. coli* (Shimizu et al., 2009; Xu et al., 2012). *Salmonella* Typhi is known to produce a specific toxin, CDT (Galan, 2016), but no candidate toxin genes have been identified in the genome of ST313 strain D23580. It is possible that BTP1-mediated lysis causes increased release of cellular material such as endotoxin, and this deserves further investigation.

Examples of prophages enhancing bacteria fitness by expressing advantageous accessory genes or by mediating spontaneous lysis are numerous (Fortier and Sekulovic, 2013; Nanda et al., 2015). However, the inevitable result of spontaneous induction of tailed phages is cell death, and therefore any benefit of lysogenic conversion or spontaneous induction itself is offset by the negative impact on bacterial growth dynamics. The high level of spontaneous BTP1 induction equates to the lysis of ∼0.2% of the bacterial population.

Therefore, it seems likely that if BTP1-mediated cell death was deleterious, ST313 derivatives containing mutations in the BTP1 prophage would have been positively selected (Lawrence et al., 2001; Bobay et al., 2014). In fact, the BTP1 prophage is highly conserved in ST313 from all over Africa (Okoro et al., 2015). This leaves the intriguing possibility that phage-mediated lysis could be tolerated if the benefit of lysogenic conversion factors such as immunity from BTP1-like phages and cargo loci were advantageous enough to outweigh the cost of spontaneous lysis. The functional conservation of BTP1 across all African ST313 isolates of both described lineages supports the hypothesis that the high rate of BTP1 spontaneous induction benefits the pathogen in some way. The impact of BTP1-mediated lysis on ST313 growth dynamics, ecological niche, virulence and fitness will be an important topic for further study.

Despite this, if there was a strong selection pressures to retain the accessory functions of BTP1, mutations could arise that inactivate prophage induction whilst retaining the lysogenic conversion phenotype, such as the mutations we report for the defective Gifsy-2, ST64B and Gifsy-1 prophages of ST313. The finding that the non-ST313-specific prophages Gifsy-1, ST64B and Gifsy-2 are defective in D23580 is intriguing. Although these prophages are present in many sequenced *S.* Typhimurium complete genomes, to our knowledge this is the first report of mutational inactivation of all three prophages in the same chromosome. However, the significance of these inactivations remains unclear.

From a phage perspective, it could be argued that temperate phages exploit their capacity to transmit either horizontally (i.e., lytically as a virion) or vertically (i.e., lysogenically as a prophage) as a bet-hedging strategy to improve overall transmission. In fact, it was recently shown that during an active infection, phage P22 regularly engages in an asymmetrically segregating phage carrier state that results in the cultivation of a transiently P22 resistant subpopulation of host cells that can subsequently be lytically consumed (Cenens et al., 2013, 2015). As such, P22 likely manages to maintain an active infection with high virion titers in a host population that is progressively taken over by P22 lysogens. In the same bet-hedging context, the BTP1 prophage could have evolved to vastly increase its spontaneous induction rate in order to insure high virion titers that increase its chance of horizontal transmission whenever the BTP1 lysogen would encounter a niche with susceptible host cells. Furthermore, such BTP1 mediated lysis of susceptible competitors would likely contribute to the ecological fitness of BTP1 lysogens as well, as was demonstrated previously with Gifsy-1 and Gifsy-2 lysogens (Bossi et al., 2003).

In summary, this thorough characterization of the prophage repertoire of *S.* Typhimurium ST313 poses interesting questions about the potential fitness costs and benefits of novel prophages in epidemic *S.* Typhimurium ST313. The high rate of BTP1-mediated spontaneous lysis represents novel biology in this clinically important sequence type, and may modulate the behavior of the pathogen in terms of ecological niche, host range, or invasiveness in humans.

## Author Contributions

Conceived and designed experiments: SO, NW, RC, MG, AA, and JH. Completed experiments and collected data: SO, NW, RC, AM, and DH. Analyzed and interpreted data: SO, NW, AA, NF, and JH. Wrote, critically revised and approved the final version of the manuscript: SO, NW, RC, AM, DH, MG, AA, NF, and JH.

## Conflict of Interest Statement

The authors declare that the research was conducted in the absence of any commercial or financial relationships that could be construed as a potential conflict of interest.

## Footnotes

1 http://www.ebi.ac.uk/ena/data/view/LT714109

2 http://enterobase.warwick.ac.uk

3 http://www.ebi.ac.uk/ena/data/view/LT714109
