# Supplementary material for: Characterization of the Prophage Repertoire of African Salmonella Typhimurium ST313 Reveals High Levels of Spontaneous Induction of Novel Phage BTP1
Source: Front Microbiol. 2017 Feb 23;8:235. doi: 10.3389/fmicb.2017.00235 (PMC5322425; doi:10.3389/fmicb.2017.00235)
Supplement: Supplementary file 1 [file Table_1.pdf]

Supplementary Material

Characterization of the Prophage Repertoire of African Salmonella Typhimurium ST313 Reveals High Levels of Spontaneous Induction of Novel Phage BTP1

Siân V. Owen, Nicolas Wenner, Rocío Canals, Angela Makumi, Disa L. Hammarlöf, Melita A. Gordon, Abram Aertsen, Nicholas A. Feasey and Jay C. D. Hinton*

*** Correspondence:** Corresponding Author: jay.hinton@liverpool.ac.uk

Supplementary Table S1: Primers used in this study

**Oligo name**	**Sequence (5'→3')**[a]	**Purposes**
BTP5_int_fw	GAACGATGCGCCAATACCAC	Amplification of a 260 bp product in BTP5 lysogenic strains
BTP5_int_rv	CATGCGTGCCGGTTAATGAG	
BTP5_attB_fw	CGGCTGGATTACAGCGTAAA	Amplification of a 167 bp if attachment site empty. No product if occupied
BTP5_attB_rv	AGGCCACTCTTTAGAGTGGC	
NW_1	ACGTGAATTCGCTGGTGCCGACGAACCATG (*Eco*RI)	Amplification of *attB*$^{Gifsy-2}$ from MA6684, pNAW16 construction
NW_4	AGCTGGATCCAACGTACAGTTCTAATGCG (*Bam*HI)	
NW_26	ACGTGAATTCCAATGATTAAAGAGTGGGC (*Eco*RI)	Amplification of *attB*$^{Gifsy-1}$ from MA6684, pNAW15 construction
NW_29	ACGTGGATCCAGGATATCTTTAATGGCGC (*Bam*HI)	
NW_52	ACGTGAATTCTAGCAGCGCAGACATCGCC (*Eco*RI)	Amplification of *attB*^{BTP1} from 4/74, pNAW17 construction
NW_53	ACGTGGATCCGAAGGCTGGCTTTATCTGGC (*Bam*HI)	
NW_54	ACGTGAATTCATCAGGCCATGCGCAAGCG (*Eco*RI)	Amplification of *attB*^{BTP5} from 4/74, pNAW18 construction
NW_55	ACGTGGATCCCTGGGCTTTGACGATCAC (*Bam*HI)	
NW_61	GCGGGTTATAACGGCGATG	*galE496* sequencing
NW_82	GAAAAGAATTCTCAGTTCCGTCAAGTTC (*Bam*HI)	
NW_86	GTCGGGGCGCTCGCCAGGAAGCTACTCTCGCGTAAAAAG ACTGACATATGAATATCCTCCTTAG	Insertion of *aph* in *STM0777-galE* intergenic region
NW_87	AGGCGCGTTACGCGCCTGGGAATGAAACGGACGGGGCAA GAGCTGTGTAGGCTGGAGCTGCTTC	
NW_161	ACTTCGGAATAGGAACTTCAAGATCCCCTAGGGATAACAG GGTAATCACGCTGCCGCAAGCACTCAGGGCGC (*I-Sce*I)	Construction of pKD4-*I-Sce*I
NW_162	GCGCCCTGAGTGCTTGCGGCAGCGTGATTACCCTGTTATCC CTAGGGGATCTTGAAGTTCCTATTCCGAAGT (*I-Sce*I)	
NW_179	ACGTGAATTCTCGATACGCTGCTCAATGTAG (*Eco*RI)	Amplification of *attB*^{ST64B} from LT2, pNAW42 construction
NW_180	ACGTGGATCCAACATTCAGCAGGGTCGGTG (*Bam*HI)	
NW_194	GCCAGAGCTTTGCCAGCCG	Modification of $P_{dinI-gfoA}$ in D23580, construction of JH3987
NW_195	GACGGATGGTTAAGTTGCAG	
NW_196	GGTTATGTAGCTTCTCTATGC	$P_{dinI-gfoA}$ sequencing in JH3987
NW_197	GCAGGATGCTCTGTATACG	
NW_214	ATTCTCATAATTCCCTCTACATTTAACTACTGTATATAAAC ACTAGGGATAACAGGGTAATC (I-SceI)	Modification of $P_{dinI-gfoA}$ in D23580, construction of JH3987
NW_215	GCAAAAAGTGCTATTCACCTCTTGAATATTCTTTCTAACAG GTATCATTTCGAACCCCAGAGTCC	
pid_Fw	GCCCAAATCGCCGCTTGC	Amplification of 380 bp product in BTP1 lysogenic strains
pid_Rev	GATTATTGTTGCGTGCCC	
RecA_pKD13_Fw	GTACGAATTCACTATCCGGTTCAATACCAAGTTGCATGACAGGAGTA ATAGTGTAGGCTGGAGCTGCTTC	Construction of LT2 ΔrecA
RecA_pKD13_Rev	GTTTTGCTGAATGGCGGCTTCGTTTTGCCCGCCCCACCATCACCTGA TGACATATGAATATCCTCCTTAG	

[a] Relevant restriction sites are underlined and indicated in brackets
