# Supplementary material for: Characterization of the Prophage Repertoire of African Salmonella Typhimurium ST313 Reveals High Levels of Spontaneous Induction of Novel Phage BTP1
Source: Front Microbiol. 2017 Feb 23;8:235. doi: 10.3389/fmicb.2017.00235 (PMC5322425; doi:10.3389/fmicb.2017.00235)
Supplement: Supplementary file 2 [file Table_2.pdf]

# *Supplementary Material*

# Characterization of the Prophage Repertoire of African Salmonella Typhimurium ST313 Reveals High Levels of Spontaneous Induction of Novel Phage BTP1

**Siân V. Owen, Nicolas Wenner, Rocío Canals, Angela Makumi, Disa L. Hammarlöf, Melita A. Gordon, Abram Aertsen, Nicholas A. Feasey and Jay C. D. Hinton***

*** Correspondence:** Corresponding Author: jay.hinton@liverpool.ac.uk

**Supplementary Table S2: Construction of suicide plasmids.** PCR amplifications of the *attB* sequences for the construction of the suicide plasmids used to cure the prophages of *S.* Typhimurium D23580

| Prophage *attB* | Primers Forward / Reverse | *S*. Typhimurium strain used as template | Restriction sites used | Insert size | Resulting pEMG derivative suicide plasmid |
|---|---|---|---|---|---|
| Gifsy-2 $^{D23580}$ (BTP2) | NW_1 / NW_4 | MA6684 | *Eco*RI / *Bam*HI | 1517 bp | **pNAW16** (pEMG::*attB*$^{Gifsy\text{-}2}$) |
| Gifsy-1 $^{D23580}$ (BTP4) | NW_26 / NW_29 | MA6684 | *Eco*RI / *Bam*HI | 1528 bp | **pNAW15** (pEMG::*attB*$^{Gifsy\text{-}1}$) |
| BTP1 | NW_52 / NW_53 | 4/74 | *Eco*RI / *Bam*HI | 1456 bp | **pNAW17** (pEMG::*attB*$^{BTP1}$) |
| BTP5 | NW_54 / NW_55 | 4/74 | *Eco*RI / *Bam*HI | 1490 bp | **pNAW18** (pEMG::*attB*$^{BTP5}$) |
| ST64B $^{D23580}$ (BTP3) | NW_179 / NW_180 | LT2 | *Eco*RI / *Bam*HI | 1521 bp | **pNAW42** (pEMG::*attB*$^{ST64B}$) |
