# Supplementary material for: Characterization of the Prophage Repertoire of African Salmonella Typhimurium ST313 Reveals High Levels of Spontaneous Induction of Novel Phage BTP1
Source: Front Microbiol. 2017 Feb 23;8:235. doi: 10.3389/fmicb.2017.00235 (PMC5322425; doi:10.3389/fmicb.2017.00235)
Supplement: Supplementary file 3 [file Table_3.pdf]

frontiers

Supplementary Material

Characterization of the Prophage Repertoire of African Salmonella Typhimurium ST313 Reveals High Levels of Spontaneous Induction of Novel Phage BTP1

Siân V. Owen, Nicolas Wenner, Rocío Canals, Angela Makumi, Disa L. Hammarlöf, Melita A. Gordon, Abram Aertsen, Nicholas A. Feasey and Jay C. D. Hinton*

*** Correspondence:** Corresponding Author: jay.hinton@liverpool.ac.uk

Supplementary Table S3. *att* core sequences for the BTP1 and BTP5 prophages. Sequences were identified based on sequence alignment of the terminal ends of the prophages

**Site**	**Sequence**
BTP1 *att* core	ATTCGTAATGCGAAGGTCGTAGGTTCGACTCCTATTATCGGCACCA
BTP5 *att* core	ACTCATAATCGCTTGGTCGCTGGTTCAAGTCCAGCAGGGGCCACCAAA TTTTAGCTTTAAAATCATATAATTAAGCCACTCTA
