# Supplementary material for: Characterization of the Prophage Repertoire of African Salmonella Typhimurium ST313 Reveals High Levels of Spontaneous Induction of Novel Phage BTP1
Source: Front Microbiol. 2017 Feb 23;8:235. doi: 10.3389/fmicb.2017.00235 (PMC5322425; doi:10.3389/fmicb.2017.00235)
Supplement: Supplementary file 4 [file Table_4.pdf]

Supplementary Material

Characterization of the Prophage Repertoire of African Salmonella Typhimurium ST313 Reveals High Levels of Spontaneous Induction of Novel Phage BTP1

Siân V. Owen, Nicolas Wenner, Rocío Canals, Angela Makumi, Disa L. Hammarlöf, Melita A. Gordon, Abram Aertsen, Nicholas A. Feasey and Jay C. D. Hinton*

*** Correspondence:** Corresponding Author: jay.hinton@liverpool.ac.uk

Supplementary Table S4. Accession numbers and location (for prophage sequences) of phages and prophages used in analysis for Figure 2. *Salmonella* prophage sequences (Casjens and Thuman-Commike, 2011)

Phage/prophage	Accession	Location (if prophage)
ST104	AB102868	N/A
P22	AF217253	N/A
ST64T	AY052766	N/A
phi20	GQ422450	N/A
g341c	FJ000341	N/A
ε34	EU570103	N/A
SE1	DQ003260	N/A
Para1	CP000026	2486731.. 2527717
Para2	FM200053	2482214.. 2523146
ParaC1	CP000857	1334931..1373190
Sari1	CP000880	2561291..2600036
Scho1	AE017220	372787..413071
Sdub1	CP001144	616909..662728
Shei1	CP001120	376852..415852
Sf6	AF547987	N/A
HK620	AF335538	N/A
CUS-3	CP000711	N/A
