# Supplementary material for: Characterization of the Prophage Repertoire of African Salmonella Typhimurium ST313 Reveals High Levels of Spontaneous Induction of Novel Phage BTP1
Source: Front Microbiol. 2017 Feb 23;8:235. doi: 10.3389/fmicb.2017.00235 (PMC5322425; doi:10.3389/fmicb.2017.00235)
Supplement: Supplementary file 5 [file Table_5.pdf]

Supplementary Material

Characterization of the Prophage Repertoire of African Salmonella Typhimurium ST313 Reveals High Levels of Spontaneous Induction of Novel Phage BTP1

Siân V. Owen, Nicolas Wenner, Rocío Canals, Angela Makumi, Disa L. Hammarlöf, Melita A. Gordon, Abram Aertsen, Nicholas A. Feasey and Jay C. D. Hinton*

*** Correspondence:** Corresponding Author: jay.hinton@liverpool.ac.uk

Supplementary Table S5. Accession numbers and location (for prophage sequences) of phages and prophages used in analysis for Figure 2B.

Phage/prophage	Accession	Host	Location (if prophage)
FLS SP-004	KC139521	*Salmonella*	N/A
SEN1	KT630644	*Salmonella*	N/A
SJ46	KT630644	*Salmonella*	N/A
SNew1	CP010283	*Salmonella* Newport str. CVM 21550	314778..375046
SWel1	LN890520	*Salmonella* Weltevreden C2346	3016958..3005246
Bong1	FR877557	*Salmonella bongori* NCTC 12419	3084587..3115233
SInf1	LN649235	*Salmonella* Infantis SINFA	4153080..4144036
sopEphi	FQ312003	*Salmonella* Typhimurium SL1344	2855616..2901979
Fels-2	AE006468	*Salmonella* Typhimurium LT2	2844421..2879237
PsP3	AY135486	Enterobacteriaceae	N/A
186	U32222	Enterobacteriaceae	N/A
L-413C	AY251033	Enterobacteriaceae	N/A
fiAA91-ss	KF322032	Enterobacteriaceae	N/A
P2	AF063097	Enterobacteriaceae	N/A
Ecol 1	CP012625	*E. coli* SF-468	1284851..1324799
Koxy 1	CP017928	*Klebsiella oxytoca* CAV1015	3759203..3793174
Smarc 1	AP013063	*Serratia marcescens* SM39	4765244..4797649
Eaer 1	FO203355	*Enterobacter aerogenes* EA1509E	5178927..5187856
