# Supplementary material for: Characterization of the Prophage Repertoire of African Salmonella Typhimurium ST313 Reveals High Levels of Spontaneous Induction of Novel Phage BTP1
Source: Front Microbiol. 2017 Feb 23;8:235. doi: 10.3389/fmicb.2017.00235 (PMC5322425; doi:10.3389/fmicb.2017.00235)
Supplement: Supplementary file 6 [file Table_6.pdf]

Supplementary Table S1. Conservation of Gifsy-2, ST64B and Gifsy-1 deactivating mutations in lineage I and II ST313 genomes. Coloured blocks indicate conservation of the mutations identified in D23580.

Strain name	Accession numbe	Lineage	Gifsy-2 mutation	ST64B mutation	Gifsy-1 mutation
A018	ERR023766	I			
C2167	ERR023767	I			
C2364	ERR023768	I			
A13198	ERR023769	I			
A082	ERR023770	I			
A357	ERR023771	I			
A680	ERR023773	I			
A3800	ERR023774	I			
A4283	ERR023775	I			
A4447	ERR023776	I			
C2110	ERR023777	I			
A13212	ERR023779	I			
A130	ERR023781	I			
D11578	ERR023783	I			
A16083	ERR023784	I			
D15040	ERR023786	I			
D25646	ERR034065	I			
D25248	ERR034071	I			
D22889_2	ERR034168	I			
O175f4	ERR038039	I			
D26104	ERR064840	I			
D15132	ERR023620	II			
D18791	ERR023621	II			
D19828	ERR023622	II			
D15176	ERR023624	II			
D15759	ERR023625	II			
C5158	ERR023626	II			
D16287	ERR023631	II			
D36099	ERR023633	II			
D37712	ERR023634	II			
D37905	ERR023635	II			
D37601	ERR023636	II			
D36225	ERR023637	II			
D36435	ERR023638	II			
D36457	ERR023639	II			
D36233	ERR023642	II			
D36448	ERR023643	II			
A16802	ERR023650	II			
A19741	ERR023653	II			
A22804	ERR023654	II			
A50063	ERR023655	II			
A50070	ERR023656	II			
A50315	ERR023657	II			
D23769a	ERR023780	II			
A38589	ERR023782	II			
D14916	ERR023785	II			
A24910	ERR023788	II			
A32751	ERR023789	II			
A32773	ERR023790	II			
A38596	ERR023792	II			
C13184	ERR023796	II			
A39051	ERR023797	II			
A39129	ERR023798	II			
A39155	ERR023799	II			
CO104	ERR024398	II			
D25023	ERR034069	II			
D25734	ERR034070	II			
D26275	ERR034072	II			
A20_2	ERR034160	II			
A40_2	ERR034161	II			
P30_2	ERR034162	II			
A21_2	ERR034163	II			
D71_2	ERR034164	II			
D83_2	ERR034165	II			
O134A	ERR037587	II			
O367A	ERR037588	II			
O363A	ERR037589	II			
O340A	ERR037590	II			
O285A	ERR037592	II			
O255A	ERR037594	II			
O175A	ERR037595	II			
O363f3	ERR038033	II			
O340f4	ERR038034	II			
O303f5	ERR038035	II			
O285f5	ERR038036	II			
O258f4	ERR038037	II			
O255f4	ERR038038	II			
O18f3s	ERR038041	II			
O134f6	ERR038042	II			
O367f2	ERR038043	II			
A54560	ERR044728	II			
A54285	ERR044729	II			
D24545	ERR064836	II			
D24871	ERR064837	II			
D25352	ERR064838	II			
D23005	ERR064839	II			
ILBSalm5409907	ERR235134	II			
ILBSalm5409909	ERR235136	II			
ILBSalm5409916	ERR235143	II			
ILBSalm5409917	ERR235144	II			
ILBSalm5409926	ERR235153	II			
ILBSalm5409937	ERR235164	II			
ILBSalm5409958	ERR235185	II			
ILBSalm5409970	ERR235197	II			
ILBSalm5409971	ERR235198	II			
ILBSalm5409972	ERR235199	II			
ILBSalm5409973	ERR235200	II			
ILBSalm5409974	ERR235201	II			
ILBSalm5409975	ERR235202	II			
ILBSalm5409976	ERR235203	II			
ILBSalm5409977	ERR235204	II			
ILBSalm5409978	ERR235205	II			
ILBSalm5409981	ERR235208	II			
ILBSalm5409983	ERR235210	II			
ILBSalm5409984	ERR235211	II			
ILBSalm5409985	ERR235212	II			
ILBSalm5409986	ERR235213	II			
ILBSalm5409989	ERR235216	II			
ILBSalm5409990	ERR235217	II			
ILBSalm5409991	ERR235218	II			
ILBSalm5409992	ERR235219	II			
ILBSalm5409994	ERR235221	II			
ILBSalm5409997	ERR235224	II			
ILBSalm5409998	ERR235225	II			
ILBSalm5409999	ERR235226	II			
ILBSalm5410000	ERR235227	II			
ILBSalm5410001	ERR235228	II			
ILBSalm5410002	ERR235229	II			
ILBSalm5410005	ERR235231	II			
ILBSalm5410006	ERR235232	II			
ILBSalm5410007	ERR235233	II			
ILBSalm5410008	ERR235234	II			
ILBSalm5410009	ERR235235	II			
ILBSalm5410010	ERR235236	II			
ILBSalm5410011	ERR235237	II			
ILBSalm5410012	ERR235238	II			
ILBSalm5410013	ERR235239	II			
ILBSalm5410014	ERR235240	II			
ILBSalm5410015	ERR235241	II			
ILBSalm5410016	ERR235242	II			
ILBSalm5410017	ERR235243	II			
ILBSalm5410018	ERR235244	II			
ILBSalm5410021	ERR235246	II			
ILBSalm5410022	ERR235247	II			
ILBSalm5410023	ERR235248	II			
ILBSalm5410024	ERR235249	II			
ILBSalm5410025	ERR235250	II			
ILBSalm5410026	ERR235251	II			
ILBSalm5410027	ERR235252	II			
ILBSalm5410028	ERR235253	II			
ILBSalm5410029	ERR235254	II			
ILBSalm5410030	ERR235255	II			
ILBSalm5410031	ERR235256	II			
ILBSalm5410032	ERR235257	II			
ILBSalm5410033	ERR235258	II			
ILBSalm5410034	ERR235259	II			
ILBSalm5410035	ERR235260	II			
ILBSalm5410036	ERR235261	II			
ILBSalm5410038	ERR235263	II			
ILBSalm5410039	ERR235264	II			
ILBSalm5410041	ERR235266	II			
ILBSalm5410044	ERR235269	II			
ILBSalm5410045	ERR235270	II			
ILBSalm5410046	ERR235271	II			
ILBSalm5410048	ERR235273	II			
ILBSalm5410049	ERR235274	II			
ILBSalm5410051	ERR235276	II			
ILBSalm5410054	ERR235279	II			
ILBSalm5410056	ERR235281	II			
ILBSalm5410057	ERR235282	II			
ILBSalm5410058	ERR235283	II			
ILBSalm5410060	ERR235285	II			
ILBSalm5410062	ERR235286	II			
ILBSalm5410063	ERR235287	II			
ILBSalm5410064	ERR235288	II			
ILBSalm5410065	ERR235289	II			
ILBSalm5410140	ERR235363	II			
ILBSalm5410141	ERR235364	II			
ILBSalm5410142	ERR235365	II			
ILBSalm5410143	ERR235366	II			
ILBSalm5410144	ERR235367	II			
ILBSalm5410145	ERR235368	II			
ILBSalm5410149	ERR235372	II			
ILBSalm5410150	ERR235373	II			
ILBSalm5410151	ERR235374	II			
ILBSalm5410152	ERR235375	II			
ILBSalm5410154	ERR235377	II			
ILBSalm5410155	ERR235378	II			
ILBSalm5410156	ERR235379	II			
ILBSalm5410158	ERR235381	II			
ILBSalm5410159	ERR235382	II			
D23580	FN424405	II			
