# Supplementary material for: Characterization of the Prophage Repertoire of African Salmonella Typhimurium ST313 Reveals High Levels of Spontaneous Induction of Novel Phage BTP1
Source: Front Microbiol. 2017 Feb 23;8:235. doi: 10.3389/fmicb.2017.00235 (PMC5322425; doi:10.3389/fmicb.2017.00235)
Supplement: Supplementary file 7 [file Image_1.pdf]

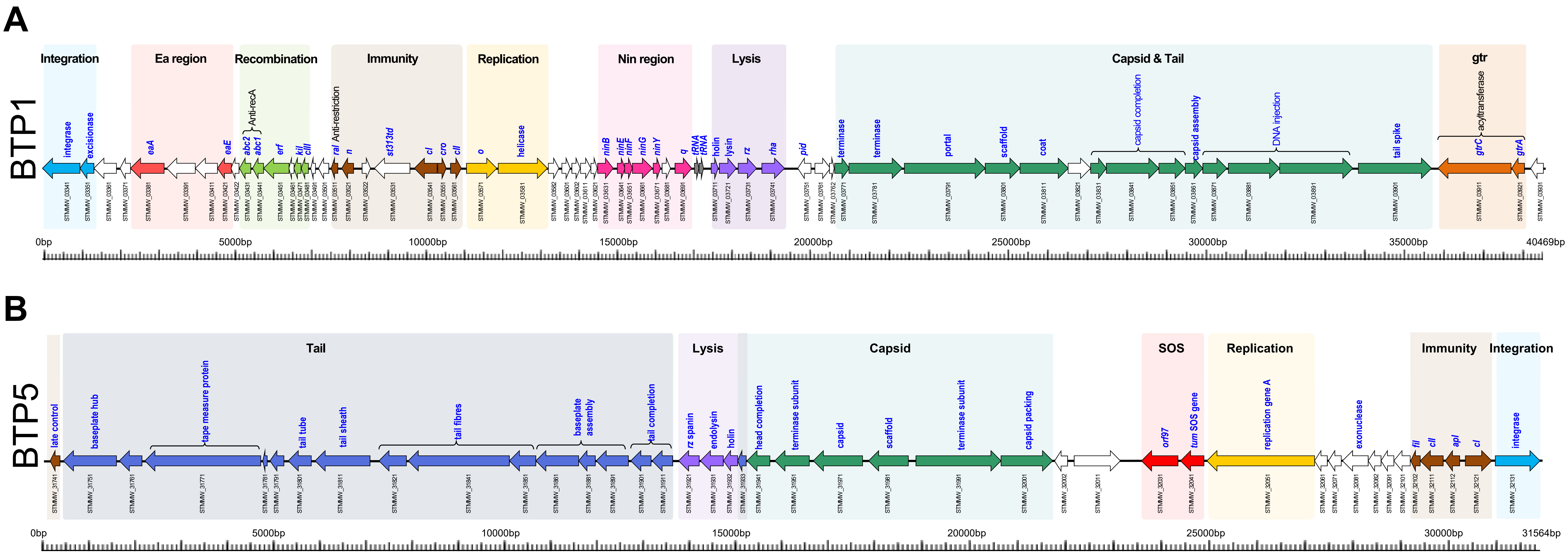

Supplementary Figure S1. Functionally annotated map of genes in prophage (A) BTP1 and (B) BTP5. Gene annotation is based on nomenclature from the lambda phage for consistency. STMMW identifiers refer to gene annotation of the D23580 genome annotation (accession FN424405).
