# Supplementary material for: Characterization of the Prophage Repertoire of African Salmonella Typhimurium ST313 Reveals High Levels of Spontaneous Induction of Novel Phage BTP1
Source: Front Microbiol. 2017 Feb 23;8:235. doi: 10.3389/fmicb.2017.00235 (PMC5322425; doi:10.3389/fmicb.2017.00235)
Supplement: Supplementary file 9 [file Image_3.pdf]

Supplementary Material

Characterization of the Prophage Repertoire of African Salmonella Typhimurium ST313 Reveals High Levels of Spontaneous Induction of Novel Phage BTP1

Siân V. Owen, Nicolas Wenner, Rocío Canals, Angela Makumi, Disa L. Hammarlöf, Melita A. Gordon, Abram Aertsen, Nicholas A. Feasey and Jay C. D. Hinton*

*** Correspondence:** Corresponding Author: jay.hinton@liverpool.ac.uk

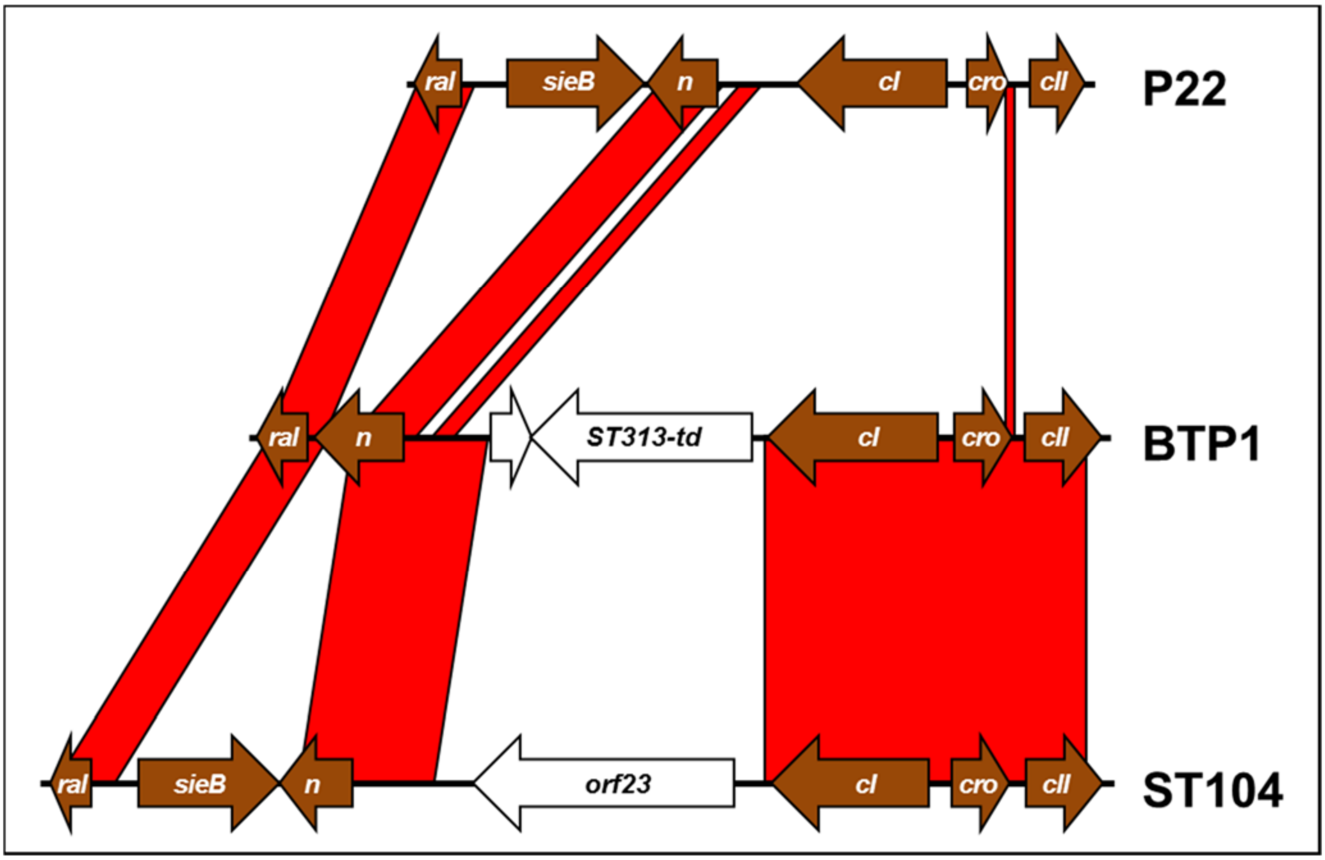

Supplementary Figure S3. Comparison of nucleotide similarity (computed using BLASTN) of the immunity regions of phage P22, BTP1 and ST104. Red blocks indicate individual BLASTN hits. For clarity genes in each immunity cluster have been annotated in accordance with lambda phage nomenclature.
